# A Case of Early Calciphylaxis Diagnosed by Bone Scan

**DOI:** 10.1155/2020/9526836

**Published:** 2020-03-17

**Authors:** Jia Di, Yuqiu Liu, Dongyan Wang, Min Yang

**Affiliations:** ^1^Nephrology, Department of Nephrology, Changzhou First People's Hospital, Changzhou, China; ^2^Institute of Nephrology, Institute of Nephrology, Zhong Da Hospital, Southeast University, School of Medicine, Nanjing, Jiangsu, China

## Abstract

Calcium uremic aortic disease (calciphylaxis) has long been considered as a rare, life-threatening small vessel disease. The diagnosis of calciphylaxis depends mainly on clinical symptoms and high risk factors, and skin biopsy can be used to confirm the diagnosis. However, noninvasive testing methods are still the focus of exploration currently. There is increasing evidence that bone scintigraphy is helpful in the diagnosis of calciphylaxis, especially for assessing the involvement of muscles and internal organs. Here, we describe a pathology-proven case of calciphylaxis case and the corresponding imaging findings on Tc-99 m MDP bone scan imaging.

## 1. Introduction

Calciphylaxis is a rare condition characterized by calcific uremic microangiopathy mainly seen in patients with end-stage renal disease (ESRD) and a small number of non-ESRD patients. As a rare, life-threatening small vessel disease, calciphylaxis is characterized by mesenteric calcification of systemic arterioles, which can lead to ischemia and subcutaneous necrosis. This disease is associated with significant morbidity and mortality with a 1-year mortality rate as high as 80% in patients with skin ulceration. Unfortunately, clinicians lack experience in early understanding of the disease, relying mainly on risk factors and typical symptoms. This case report presents a patient with ESRD who was initially diagnosed with calcification after bone scintigraphy and at last pathologically proved.

## 2. Case Presentation

**Figure 1:**
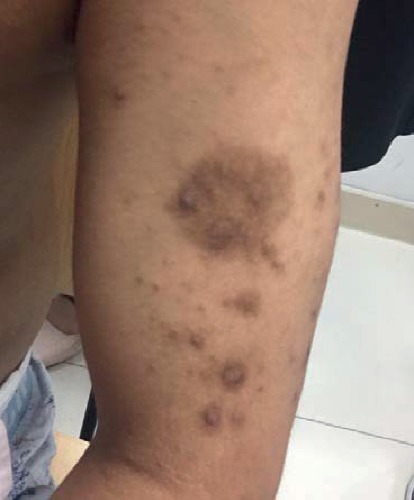
Multiple gray-white granular nodules present on the patient's skin.

**Figure 2:**
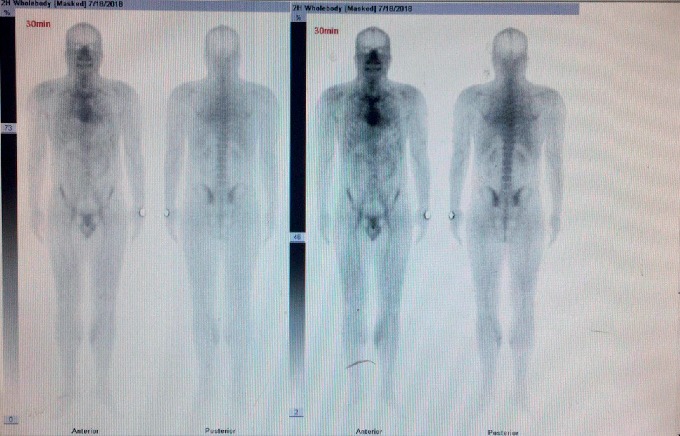
Angiographic flow phase images (blood-pool phase, 0h) showing asymmetric perfusion in the lower extremities.

**Figure 3:**
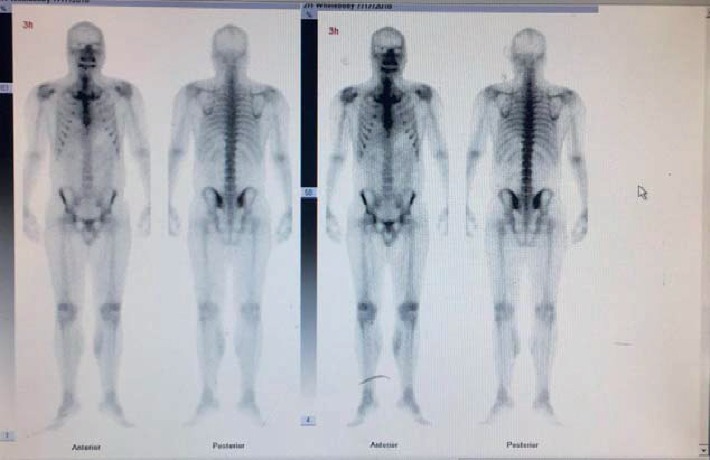
Delayed images (blood-pool phase, 3h) showing moderate-to-intense uptake related to superficial soft tissues of the left calf muscles.

**Figure 4:**
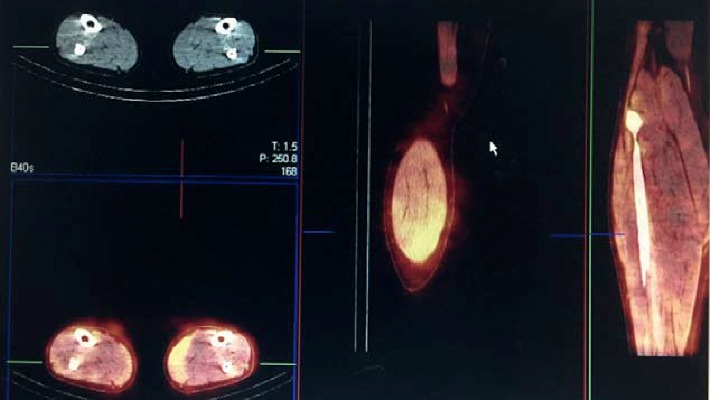
Fusion images showing the areas of abnormal radiotracer uptake were at the left lateral gastrocnemius muscle.

**Figure 5:**
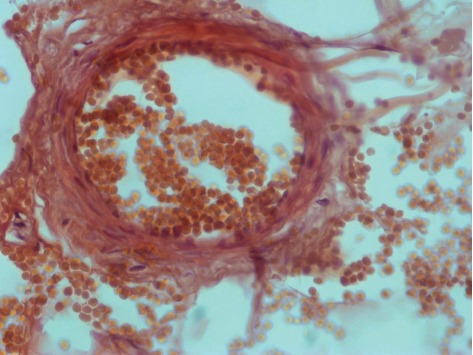
Skin biopsy showing subcutaneous arteriole calcification.

A 32-year-old male patient was diagnosed with chronic renal failure in December 2014, and his serum creatinine gradually increased later. He entered continuous ambulatory peritoneal dialysis in September 2016 (peritoneal dialysate 2000 ml qid, calcium concentration 1.5 mmol/l). More than 1 year ago, there were multiple gray-white granular nodules on the patient's skin, mainly distributed in the neck and back, without pain and itching. These nodules gradually progressed to the upper limbs and shoulders in the following six months (Figure 1). After extruding, the nodules can be partially detached, leaving pigment spots of 0.2 to 1.5 cm in size. The patient was scheduled for a metastable Technetium-99 methyl diphosphonate (99m TcMDP) bone scan, and a significant positive result was obtained. Angiographic flow phase images showed asymmetric perfusion in the lower extremities with the left greater than the right (Figure 2). Delayed images showed moderate-to-intense uptake related to superficial soft tissues of the left calf muscles (Figure 3). Fusion images showed that the areas of abnormal radiotracer uptake were at the left lateral gastrocnemius muscle (Figure 4). The patient took calcium carbonate 2-3 tablets everyday for 3 months in 2015 and Rocaltrol 0.25 g/d from September 2016 till now. He had previous history of hypertension and hepatitis B but no diabetes, without glucocorticoids, immunosuppressants, warfarin, and heparin application history. Bone scan results strongly suggest a diagnosis of calciphylaxis. For further diagnosis and treatment, skin biopsy (pea-sized tissue) was performed, and the results showed subcutaneous arteriole calcification and extensive calcium salt deposition confirming calciphylaxis (Figure 5). Then, the patient was treated with sodium thiosulfate (6.4 g/d) for 21 days, and the skin lesions were diminished.

## 3. Discussion

In this case, we describe an early calciphylaxis case with gray-white nodules as the main symptoms. Calciphylaxis is a rare, life-threatening disease with poor prognosis, and effective therapies are lacking. Calciphylaxisis is diagnosed mainly by typical lesions and risk factors. Typical lesions are presented as reticular plaques, violet plaques, and hardened nodules, locating in the skin, subcutaneous tissue, and soft and fat-rich tissue areas. But once these lesions appear, the condition is usually irreversible and rapidly deteriorated, even to death. So, identification of the early stage of calciphylaxis is very important to improve patient's prognosis. Even so, it is difficult because early symptoms are often lack of characteristic changes. Skin biopsy is the gold standard for the diagnosis of calcification; however, it can result in new nonhealing ulcers and skin infections, so its value in the diagnosis of calcification remains controversial [1]. As a final diagnostic test, skin biopsy is often considered in the diagnosis of calcification, but it is still not systematically performed due to the risk of further spreading of the lesions [2]. As the early necrotic ulcers may quickly spread or infect and result in ominous outcome, a noninvasive method would be necessary for early diagnosis and improve prognosis.

Imaging examination (X-ray examination and bone radionuclide scanning) can be helpful when pathology is not definite or taking biopsy is not feasible. Grid-like subcutaneous calcification on X-plain films and increased tracer uptake on heterogeneity with bone scans are highly specific. When calciphylaxis occurred in patients with end-stage renal disease, new bone formation is present in the soft tissue. A Tc-99 m MDP bone scan detects osteoblastic activity by chemisorption to hydroxyapatite crystals in new forming bone [3]. Using bone scintigraphy to diagnose calciphylaxis has been reported in some reports [4–7].

Calciphylaxis has been traditionally considered as a manifestation of mineral bone disorders (such as hyperphosphatemia, elevated calcium phosphorous product, hypocalcemia, hyperparathyroidism, and vitamin D deficiency) in dialysis patients. So dysregulated calcium-phosphorous metabolism is a risk factor for calciphylaxis. However, still some reports describe calciphylaxis without significant mineral bone abnormalities and require further investigation in larger observation studies [8]. In our case report, this patient has risk factors of calciphylaxis such as dialysis, taking active vitamin D, secondary hyperparathyroidism, and grayish nodules, and the Tc-99 m MDP bone scan demonstrated the areas of abnormal radiotracer uptake at his left lateral gastrocnemius muscle. Combined with skin biopsy, the diagnosis of early calciphylaxis could be established.

Based on these findings, we found that bone scintigraphy is highly sensitive and specific for the diagnosis of calciphylaxis. It is recommended to fully evaluate the imaging examinations such as bone scans for patients with suspected calciphylaxis and to find diagnostic clues from the angle of imaging.
